# Supplementary figures and images for: Automatic body morphometric analysis of adult zebrafish using microCT
Source: PLoS One. 2026 Aug 3;21(8):e0354249. doi: 10.1371/journal.pone.0354249 (PMC13432094; doi:10.1371/journal.pone.0354249)

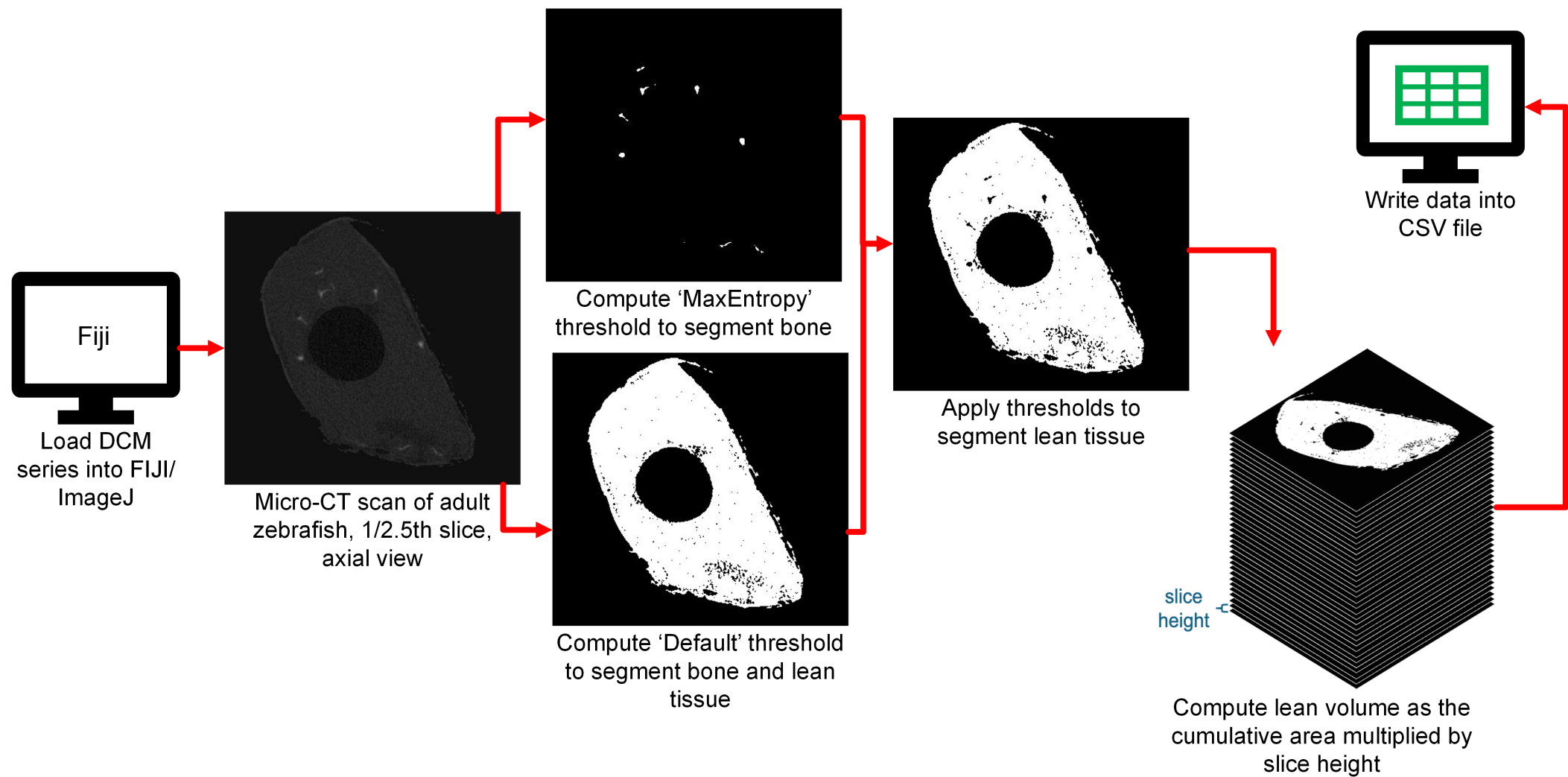

Supplement: S1 Fig — In this procedure, thresholds are automatically computed and used to segment lean tissue, and the total lean volume is determined by summing the areas of lean tissue across slices and multiplying by the slice height. (PDF) [file pone.0354249.s001.pdf]

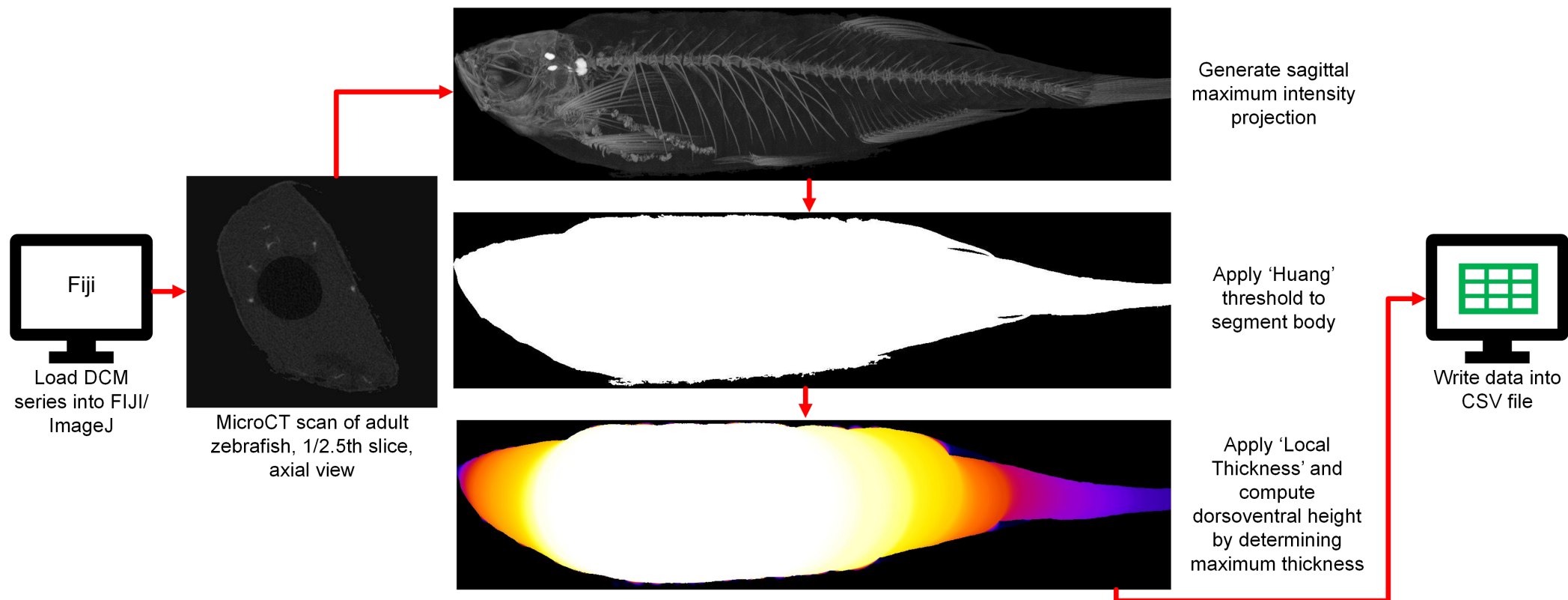

Supplement: S3 Fig — In this procedure, the fish body is segmented in a sagittal maximum intensity projection, the ‘Local Thickness’ tool is applied, and the dorsoventral height is determined as the maximum thickness. (PDF) [file pone.0354249.s003.pdf]
