# Supplementary material for: Automatic body morphometric analysis of adult zebrafish using microCT
Source: PLoS One. 2026 Aug 3;21(8):e0354249. doi: 10.1371/journal.pone.0354249 (PMC13432094; doi:10.1371/journal.pone.0354249)
Supplement: S2 Fig — In Method 1, the base of the caudal fin rays is located by analyzing sagittal maximum intensity projections. Here, after bone segmentation, the ‘Watershed Irregular Features’ tool is used to separate the caudal vertebrae from the caudal fin rays, the latter of which appear as one large island due to partial volume effects. The location of the base of the caudal fin rays is determined by the anterior-most boundary (BY) of the largest island in the region. In Method 2, the base of the caudal fin rays is located by traversing through the stack. Specifically, after bone segmentation, the ‘Analyze Particles’ tool is used to determine the size and elongation of islands in each slice; the location of the base of the caudal fin rays is determined as the slice containing the anterior-most of structures meeting a certain size and elongation. (PDF) [file pone.0354249.s002.pdf]

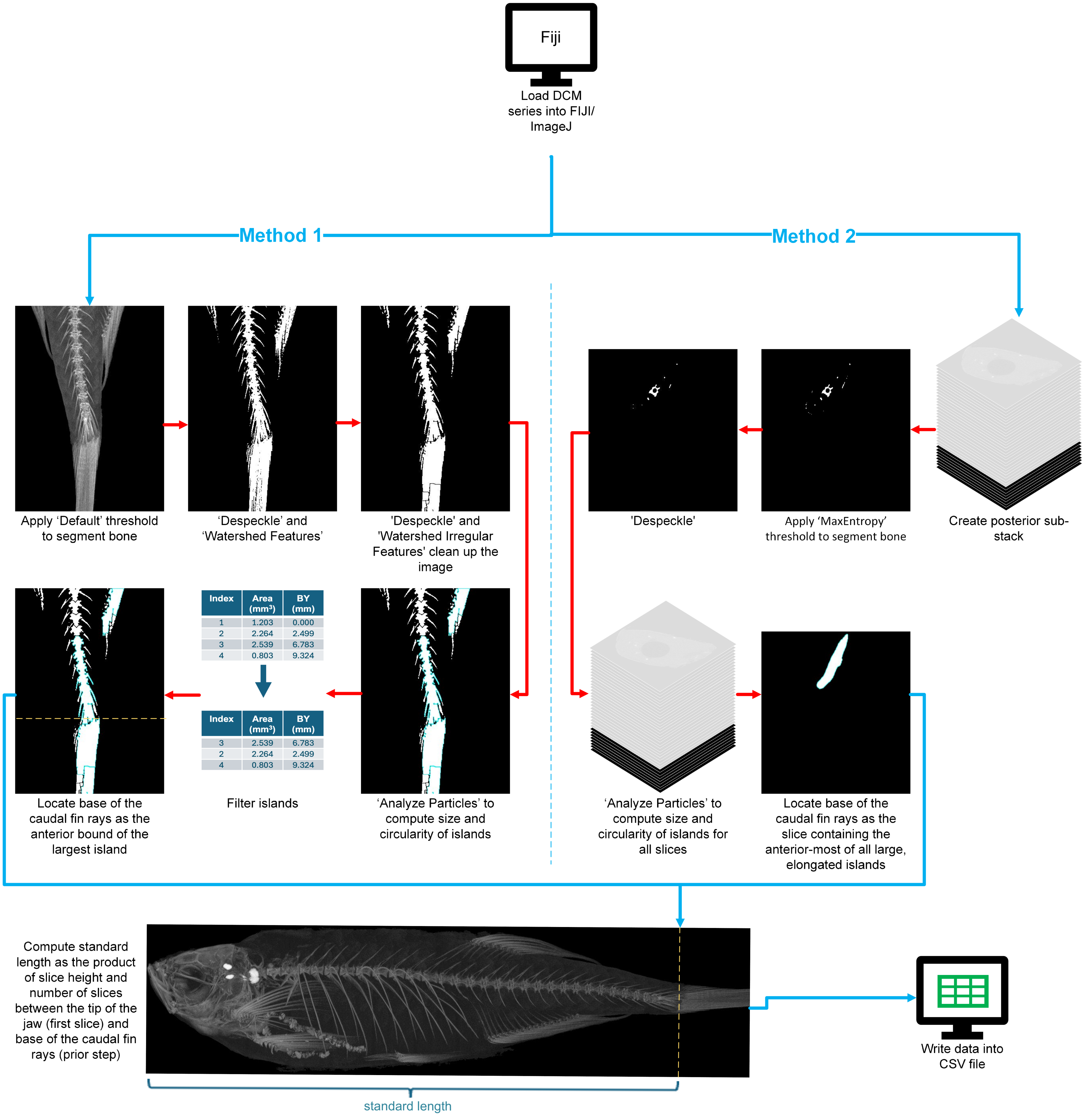

Fiji
Load DCM series into FIJI/ ImageJ
Method 1
Method 2
Apply 'Default' threshold to segment bone
'Despeckle' and 'Watershed Features'
'Despeckle' and 'Watershed Irregular Features' clean up the image
'Despeckle'
Apply 'MaxEntropy' threshold to segment bone
Create posterior sub-stack
Index | Area (mm³) | BY (mm)
1 | 1.203 | 0.000
2 | 2.264 | 2.499
3 | 2.539 | 6.783
4 | 0.803 | 9.324
Index | Area (mm³) | BY (mm)
3 | 2.539 | 6.783
2 | 2.264 | 2.499
4 | 0.803 | 9.324
Locate base of the caudal fin rays as the anterior bound of the largest island
Filter islands
'Analyze Particles' to compute size and circularity of islands
'Analyze Particles' to compute size and circularity of islands for all slices
Locate base of the caudal fin rays as the slice containing the anterior-most of all large, elongated islands
Compute standard length as the product of slice height and number of slices between the tip of the jaw (first slice) and base of the caudal fin rays (prior step)
standard length
Write data into CSV file
