# Supplementary material for: Automatic body morphometric analysis of adult zebrafish using microCT
Source: PLoS One. 2026 Aug 3;21(8):e0354249. doi: 10.1371/journal.pone.0354249 (PMC13432094; doi:10.1371/journal.pone.0354249)
Supplement: S4 Fig — Here, images are rotated, the swim bladders are segmented in a sagittal minimum intensity projection, and the anterior and posterior swim bladder lengths are computed as the major axes of the two largest islands. Results are output in a CSV file, with manual evaluation suggested for uncertain cases. (PDF) [file pone.0354249.s004.pdf]

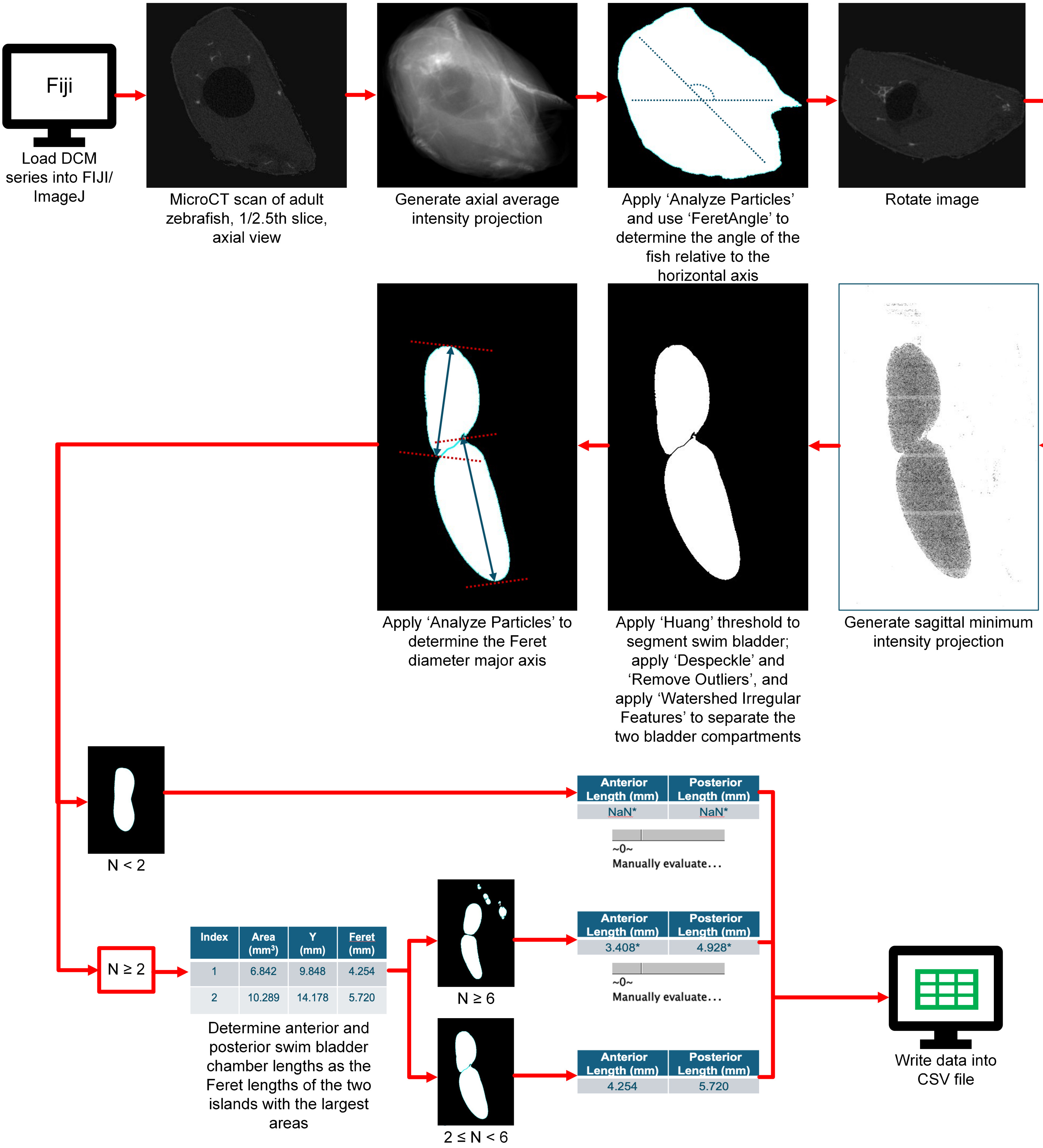
Fiji
Load DCM series into FIJI/ ImageJ
MicroCT scan of adult zebrafish, 1/2.5th slice, axial view
Generate axial average intensity projection
Apply ‘Analyze Particles’ and use ‘FeretAngle’ to determine the angle of the fish relative to the horizontal axis
Rotate image
Generate sagittal minimum intensity projection
Apply ‘Huang’ threshold to segment swim bladder; apply ‘Despeckle’ and ‘Remove Outliers’, and apply ‘Watershed Irregular Features’ to separate the two bladder compartments
Apply ‘Analyze Particles’ to determine the Feret diameter major axis
N < 2
Anterior Length (mm) | Posterior Length (mm)
NaN* | NaN*
~0~
Manually evaluate...
N ≥ 2
Index | Area (mm³) | Y (mm) | Feret (mm)
1 | 6.842 | 9.848 | 4.254
2 | 10.289 | 14.178 | 5.720
Determine anterior and posterior swim bladder chamber lengths as the Feret lengths of the two islands with the largest areas
N ≥ 6
Anterior Length (mm) | Posterior Length (mm)
3.408* | 4.928*
~0~
Manually evaluate...
2 ≤ N < 6
Anterior Length (mm) | Posterior Length (mm)
4.254 | 5.720
Write data into CSV file
